# Freda E. Martin, MD, FRCPsych, FRCPsych (Canada)

**DOI:** 10.1192/bjb.2020.7

**Published:** 2020-10

**Authors:** Dora Black

**Formerly Chair, Department for Children and Parents, Tavistock Clinic, London, and Executive Director, CM Hincks Treatment Centre, Toronto, Canada**

**Figure:**
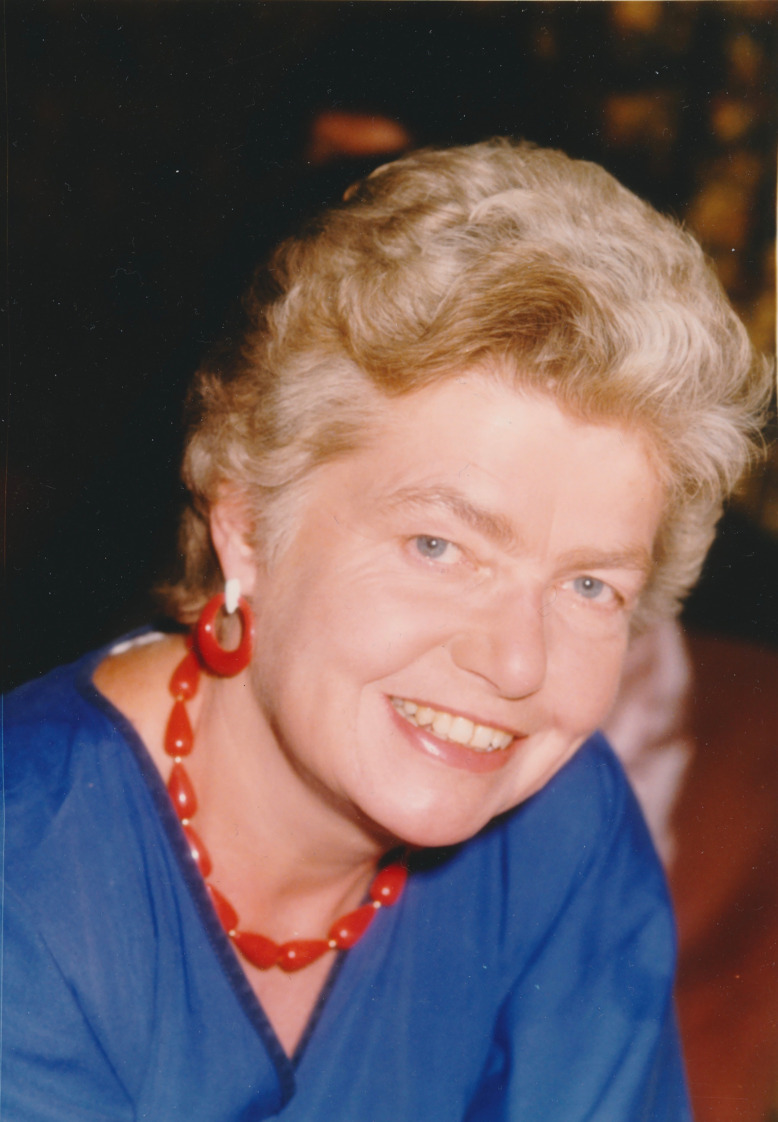

Freda Martin, who died in Toronto on 2 August 2019 at the age of 87, was one of the early pioneers of family therapy, first in the UK and then in Canada. In 1962, while working at the Tavistock Clinic, London, in collaboration with Janet Knight, she published an important paper describing the assessment of disturbed children involving the whole family.^1^ At that time, the standard assessment of such children involved the mother being seen by a social worker, while the child saw the psychiatrist. Fathers were either not seen at all or seen usually on only one occasion with the mother. Freda Martin, with Rosemary Whiffen and John Byng-Hall, influenced both by John Bowlby and the family therapy movement in the USA, was able to demonstrate how not just assessing but treating the whole family as a unit often illuminated the family dynamics in a manner impossible to achieve when family members were seen individually.

Freda came to the Tavistock Clinic with an unusual background. Arriving there in 1960 as the first psychiatrist with a Jungian, rather than a Kleinian, analytic training, she was not aligned with the existing predominantly psychoanalytic factions. This enabled her to be eclectic in her clinical work and to espouse her belief in the importance of multidisciplinary teamwork, involving psychiatrists, psychologists, social workers and psychotherapists.

Initially, she met much hostility at the Tavistock Clinic from those who found it difficult to accept that an analyst could embrace such views. She persisted, first as one of a number of colleagues and later as Chair of the Department for Children and Parents.

In 1975, she returned to Canada, eventually becoming Executive Director of the CM Hincks Treatment Centre in Toronto. Here she was able to introduce clinical approaches derived from attachment theory. According to a colleague, she brought to Canada the importance of early intervention to promote physical and emotional well-being in all children, as well as her belief in the importance of postgraduate training for all disciplines, which she regarded as equally necessary for helping children and families. Today these ideas are accepted and practised, even in centres formerly devoted to only one treatment mode for disturbed children.

She initiated postgraduate multidisciplinary training and research into what previously had been a purely clinical setting. The renamed Hincks Dellcrest Centre Gail Appel Institute, now part of the Hospital for Sick Children, Toronto, came into being in 1986. It required new, expanded premises. Freda Martin persuaded the Board to construct a seven-floor building on the clinic car park. It was opened by John Bowlby.

She continued to develop new approaches to treatment applying them, for example, to the successful treatment of anorexia in adolescents. In a seminal paper published in 1990,^2^ she described what she called ‘run-away’ effects at various systemic levels (physiological, individual, family, social), which triggered dieting behaviour in perfectionistic personalities.

Creating a link with the University of Toronto, where she was an assistant professor, enabled her to create fellowships to train social workers and psychologists, educators and psychotherapists in family therapy and infant mental health.

Freda Martin (née McQueen) was born in Niagara Falls, Canada, on 11 April 1932 to Andrew, an engineer, and Lily. She studied medicine at the University of Toronto Medical School, qualifying in 1956. In 1959, she came to the Maudsley Hospital, London, for postgraduate psychiatric training. She was appointed a senior registrar at the Tavistock Clinic in 1960, shortly afterwards becoming a consultant and then Chair of the Department for Children and Parents.

Freda met her husband Kenneth Martin, an adult psychiatrist who worked mainly in private practice, at the Maudsley. They had two children. Freda and her family loved to relax in the family ‘cottage’ by Devil Lake, where they sailed, canoed and entertained friends.

After leaving her full-time post in 2005, Martin became involved in advising the provincial and federal governments about the importance of teaching parents and educators about early physical and emotional development. She set up and evaluated 3-year ‘learning through play’ courses all over Canada. Later, she worked in Jamaica and Latvia with senior professionals using this material to train parents, carers and nursery staff. These activities were funded by the Canadian International Development Agency. After full retirement, she took singing lessons and enjoyed singing in a choir. Very recently she helped children to read as well as reading to fellow residents in her retirement home.

Freda is described by family members and her many devoted colleagues as outstandingly creative, decisive, forthright and brisk. She was not afraid to speak her mind, although she was always regarded as kind and supportive to staff and students.

The daughter of a close friend described her as a ‘bullshit detector’. When this same friend was terminally ill, Freda, in the words of her friend's daughter, ‘enabled mum to articulate and process her own death’, with support and love, helping the family in their grief.

Kenneth died in 2012. Freda is survived by her two children, Andrew and Peter, as well as by two grandchildren.
